# Probing white-matter microstructure with higher-order diffusion tensors and susceptibility tensor MRI

**DOI:** 10.3389/fnint.2013.00011

**Published:** 2013-03-06

**Authors:** Chunlei Liu, Nicole E. Murphy, Wei Li

**Affiliations:** ^1^Brain Imaging and Analysis Center, School of Medicine, Duke UniversityDurham, NC, USA; ^2^Department of Radiology, Duke UniversityDurham, NC, USA

**Keywords:** MRI, white matter, diffusion tensor imaging, generalized diffusion tensor imaging, susceptibility tensor imaging, higher order tensor, cumulant, kurtosis

## Abstract

Diffusion MRI has become an invaluable tool for studying white matter microstructure and brain connectivity. The emergence of quantitative susceptibility mapping and susceptibility tensor imaging (STI) has provided another unique tool for assessing the structure of white matter. In the highly ordered white matter structure, diffusion MRI measures hindered water mobility induced by various tissue and cell membranes, while susceptibility sensitizes to the molecular composition and axonal arrangement. Integrating these two methods may produce new insights into the complex physiology of white matter. In this study, we investigated the relationship between diffusion and magnetic susceptibility in the white matter. Experiments were conducted on phantoms and human brains *in vivo*. Diffusion properties were quantified with the diffusion tensor model and also with the higher order tensor model based on the cumulant expansion. Frequency shift and susceptibility tensor were measured with quantitative susceptibility mapping and susceptibility tensor imaging. These diffusion and susceptibility quantities were compared and correlated in regions of single fiber bundles and regions of multiple fiber orientations. Relationships were established with similarities and differences identified. It is believed that diffusion MRI and susceptibility MRI provide complementary information of the microstructure of white matter. Together, they allow a more complete assessment of healthy and diseased brains.

## Introduction

Probing the microstructure of white matter has major applications in a number of neurological diseases and disorders. The abilities to map brain connectivity *in vivo* were initially made possible because of the discovery of diffusion anisotropy in the white matter (Moseley et al., 1990) and the development of a range of techniques based on this phenomenon, most notably the development of diffusion tensor imaging (DTI) and tractography (Basser et al., 1994, 2000; Conturo et al., 1999; Mori et al., 1999). It is often reported that DTI, or more generally diffusion MRI, is the only method available for imaging white matter fiber tracts *in vivo* and non-invasively, which is a clear testament to the importance of DTI for studying brain connectivity. The recent discovery of magnetic susceptibility anisotropy (MSA) in white matter and the development of susceptibility tensor imaging (STI) may potentially provide a viable complementary method for imaging fiber tracts *in vivo* and non-invasively (Lee et al., 2010; Liu, 2010; Li et al., 2011, 2012a,b; Liu et al., 2012).

The existence of MSA has now been verified in simulation, mouse brains, specimens, and live human brains. For example, by rotating intact mouse brains in small-bore animal 7T scanner, Liu showed that the magnetic susceptibility of white matter demonstrated strong orientation dependence (Liu, 2010). This orientation dependence has also been observed by Lee et al. on segments of corpus callosums from a postmortem human brain (Lee et al., 2010). Similar to diffusion anisotropy, susceptibility anisotropy can also be described by a rank-2 tensor, the apparent susceptibility tensor (Liu, 2010). This susceptibility anisotropy and susceptibility tensor can be measured based on a simple 3D gradient-recalled-echo sequence. Variations among tissue magnetic susceptibility cause a tissue dependent frequency shift that manifests as a phase shift in gradient echo images. By measuring this frequency shift, a susceptibility tensor may be determined for each voxel of the brain. Susceptibility anisotropy and susceptibility tensors have thus far been imaged on both 3T and 7T *in vivo* (Li et al., 2011, 2012b; Schweser et al., 2012). Studies have shown that the major eigenvector orientation of the susceptibility tensor is aligned with axons in parallel (Lee et al., 2010; Liu, 2010; Li et al., 2012a,b; Liu et al., 2012). It has been reported that the apparent magnetic susceptibility (AMS) of the white matter is the most paramagnetic when the underlying axons are parallel to the magnetic field (Lee et al., 2010; Liu, 2010). It was proposed that this characteristic originates from the radially aligned myelin lipids (Li et al., 2012a). A recent study has utilized this relationship to perform fiber tracking in the mouse brain which has demonstrated similar tracks with DTI tractography in large fiber bundles (Liu et al., 2012).

An important challenge of MRI-based *in vivo* fiber tracking is the relative large voxel size (on the order of millimeters) compared to the size of axons which is on the order of micrometers. In large parallel fiber bundles, this mismatch of spatial scales is not problematic as the orientations of the axons are largely similar. However, in a large portion of the brain, each voxel of MRI images is known to contain fibers of different orientations, thus creating the situation of “crossing” or “kissing” fibers (Basser et al., 2000). Achieving high angular resolution by resolving these crossing fibers has been a major goal of diffusion based fiber tractography in the past decade (Frank, 2001, 2002; Alexander et al., 2002; Tuch et al., 2002; Lin et al., 2003; Liu et al., 2003a,b; Ozarslan and Mareci, 2003; Tuch, 2004; Jensen et al., 2005). A number of techniques have been proposed including, for example, q-space or diffusion spectrum imaging (King et al., 1994; Lin et al., 2003; Wedeen et al., 2008), q-ball imaging (Tuch, 2004) and higher order tensor models (Liu et al., 2003b, 2004; Ozarslan and Mareci, 2003) to name a few. Similarly, STI may encounter the same challenges. The behavior of magnetic susceptibility in the presence of these complex fiber architectures, however, has not been studied.

The goal of the present study was to investigate the behavior of magnetic susceptibility in the presence of fiber crossings and its relationship with higher order diffusion anisotropy. We first evaluated the variation of magnetic susceptibility as a function of magnetic field orientation in a simulated phantom of crossing fibers. The behavior was compared to that of higher order diffusion tensors calculated based on the cumulant expansion (covariance, skewness and kurtosis etc) (Liu et al., 2003a,b, 2004; Jensen et al., 2005). Experiments were also conducted *in vivo* to assess the effect of fiber crossings on magnetic susceptibility.

## Materials and methods

### Susceptibility tensor and susceptibility orientation distribution

Since the early 1990s, diffusion MRI based on diffusion anisotropy has been the only means to study the orientations and organizations of white matter fiber tracts *in vivo* and non-invasively. In the past couple of years, it has become apparent that magnetic susceptibility of the white matter also exhibits anisotropy. While the ordered arrangement of axons largely contributed diffusion anisotropy (Beaulieu, 2002; Song et al., 2002), the ordered arrangement of myelin lipids is believed to be the main source of MSA (Li et al., 2012a). Susceptibility anisotropy may be quantified with the technique of susceptibility tensor imaging. Magnetic susceptibility of the brain results in a measurable frequency shift in the gradient-echo images. By solving the Maxwell's equation, this frequency shift denoted as θ was found to be related to a spatially distributed susceptibility tensor field **χ**(**r**) following (Liu, 2010)

(1) $$\theta =FT^{-1}\left\{\frac{1}{3}{\hat{H}}^{T}FT\{\chi \}\hat{H}-\hat{H}\cdot k\frac{k^{T}FT\{\chi \}\hat{H}}{k^{2}}\right\}\gamma Ht$$

Here, *FT* and *FT*^−1^ represent Fourier and inverse Fourier transform respectively; **k** is the spatial frequency vector reciprocal to **r**; *H* is the magnitude of the applied magnetic field; **Ĥ** is the unit vector of the applied magnetic field; *t* is the echo time (TE) in a gradient echo sequence and γ is the gyromagnetic ratio.

The susceptibility tensor **χ**(**r**) is assumed to be a 3 × 3 symmetric matrix with 6 independent elements (3 diagonal and 3 off diagonal). When the demagnetization field [the second term of Equation (1)] is ignored, this symmetry property is evident as swapping the indices of **χ**(**r**) does not change θ. Given this symmetry assumption, a minimum of 6 independent measurements are needed to determine a susceptibility tensor. Each independent measurement will require the magnetic field to be oriented in a different direction with respect to the object. As an analogy to DTI, this is equivalent to changing the directions of diffusion encoding gradients. And, similar to the measurement of the diffusion tensor, the angle separation between orientations should ideally spread apart. As in any other experimental measurements, more orientations will improve the matrix condition and allow a more accurate determination of the tensor elements. Once the susceptibility tensor is calculated, it can then be decomposed into its eigensystem with the eigenvalue decomposition. One advantage of eigenvalue decomposition is that it expresses susceptibility tensor in a coordinate system that is independent of the experimental coordinate system. As a result, the values can be compared between different scans and different subjects.

The relationship between susceptibility tensor and fiber orientations has been established in the case of parallel fibers. It has been shown that the direction of the major eigenvector of the susceptibility tensor (most paramagnetic) is parallel to the direction of the axons (Lee et al., 2010; Liu, 2010). The relationship has been confirmed broadly with theory, simulation, brain specimens, *ex vivo* and *in vivo* brain imaging at 3T and 7T. In the case of fiber crossing, however, a single susceptibility tensor is unlikely to convey the existence of multiple fiber orientations. One way to address this limitation is to use multiple susceptibility tensors to characterize the susceptibility property of a voxel. Alternatively, we can plot the AMS as a function of the field orientation. The orientation variation of the AMS may depict the existence of multiple fibers. These orientations can then be compared to those based on diffusion models.

### Higher-order tensor (HOT) reconstruction

In the cumulant expansion, the diffusion-weighted MRI signal is related to a set of higher-order tensors following (Liu et al., 2003b, 2004)

(2) $$s(b)=s(0)\mathrm{exp}\left\{\sum_{n\;=\;2}^{\infty }{\left(-j\right)}^{n}D_{i_{1},i_{2},\ldots ,i_{n}}^{\left(n\right)}b_{i_{1},i_{2},\ldots ,i_{n}}^{\left(n\right)}\right\}$$

Here, *s*(*b*) is the diffusion weighted signal and *s*(0) is non-diffusion weighted signal; *j* is the imaginary number; *D*^(n)^ is the *n*-th order cumulant tensor with *n* = 2 corresponding to the covariance matrix, *n* = 3 corresponding to the skewness tensor and *n* = 4 corresponding to the kurtosis tensor; *b*(*n*) is an *n*-th order tensor describing diffusion weighting factors. The Einstein's summation rule is assumed for the subscripts of *D*^(n)^ and *b*^(n)^. It has been shown that *D*^(n)^ is a symmetric tensor as it is related to the partial differentiation of the particle concentration in Fick's second law (Liu et al., 2003b, 2004).

Although Equation (2) can be readily solved with least-squares estimations given a set of measurements at different diffusion encoding directions and *b*-values, the solution for the higher-order tensor (HOT) may be easily biased by the noise at large *b*-values. Specifically, because *s*(*b*) is Rician distributed, it does not decay to zero. Instead, it approaches the noise floor as the *b*-value increases, resulting in an overestimate of the non-Gaussianity and higher-order tensors. To reduce this overestimation, we apply Tikhonov regularization by solving the following minimization problem for a fourth-order approximation

(3) $$\underset{D^{\left(2\right)},D^{\left(4\right)}}{\min}\;{\left\|\ln(s(b)/s(0))+b_{i_{1},i_{2}}^{\left(2\right)}D_{i_{1},i_{2}}^{\left(2\right)}-b_{i_{1},i_{2},i_{3},i_{4}}^{\left(4\right)}D_{i_{1},i_{2},i_{3},i_{4}}^{\left(4\right)}\right\|}^{2}+{\left\|\Gamma_{i_{1},i_{2},i_{3},i_{4}}D_{i_{1},i_{2},i_{3},i_{4}}^{\left(4\right)}\right\|}^{2}$$

The Tikhonov matrix Γ is a diagonal fourth-order tensor with a diagonal element of

(4) $$\lambda =\text{trace}(b^{\left(4\right)}){\left(\bar{D}/D_{0}\right)}^{5}$$

Here, $\bar{D}$ is the mean diffusivity of a given voxel estimated by DTI using the smallest *b*-value; *D*_0_ is a diffusion coefficient chosen to be 1.5 × 10^−3^ mm^2^/s, slightly smaller than that of cerebral spinal fluid but larger than that of gray matter. With this choice of regularization parameter, the fourth-order tensor of the CSF is suppressed assuming the diffusion is mainly Gaussian in the CSF. By raising the ratio of $\bar{D}$ and *D*_0_ to the fifth power, the effect of regularization is reduced for the white matter. The scaling factor of trace[*b*^(4)^] is included to ensure the regularization term is dimensionless.

Coordinate system independent quantities can be defined with eigenvalue decomposition. There have been some increasing efforts to better understand the algebra of fourth-order tensors in the past decade (Miehe, 1993; Browaeys and Chevrot, 2004; Muti and Bourennane, 2005). Nevertheless, a standard of decomposition for fourth-order tensors is not fully established yet. We chose to convert the fourth-order diffusion tensor with a dimension of 3 × 3× 3 × 3 to a two-dimensional 9 × 9 matrix denoted as **T**. This technique has been widely used in describing the stiffness tensor of materials' mechanical property (Nemat-Nasser and Hori, 1999; Basser and Pajevic, 2007). This can be accomplished by transferring each *D*^(4)^_*i*_1_*i*_2_*i*_3_*i*_4__ component to element *T*_pq_. The rule of transformation between 4D index and 2D index is given in Table 1. For example, the fourth-order element *D*_1231_ is transformed to element *T*_27_ following the rule. Decomposing tensor T results in 9 eigenvalues.

**Table 1:** **Indices for unwrapping a fourth-order tensor *D*_*ijkl*_ into a 9 × 9 matrix *T*_*pq*_**.

*p* (*q*)	1	2	3	4	5	6	7	8	9
*ij* (*kl*)	11	12	13	21	22	23	31	32	33

Once the higher order diffusion tensors are measured, the probability density function (PDF) of the underlying diffusion process can be computed using the Gram-Charlier series (Liu et al., 2003b, 2004). The Gram-Charlier series is widely used expansion based on the Hermite tensor (Zucker and Schulz, 1982).

### Phantom experiments and simulation

A phantom of crossing fibers was used for diffusion experiments. The phantom consisted of sheets of parallel plastic capillaries with an inner diameter of 50 μm and an outer diameter of 350 μm (PTFE ultramicrobore tubing P-06417-70, Cole-Parmer Instrument, Vernon Hills, IL). Two sets of capillaries were overlapped at a 90° angle. Imaging was performed on a 3T MRI system with a high-performance insert gradient set. Scan parameters were: FOV = 25 × 25 cm^2^, *TR* = 1900 ms, *TE* = 13.8 ms, single slice, *NEX* = 4 and matrix size = 32 × 32. Diffusion-weighted images were acquired with a stimulated echo for 160 diffusion encoding directions and for *b*-values of 500, 1000, 2000, 4000, and 8000 s/mm^2^.

However, this phantom did not mimic the susceptibility effect of myelin structures while it is known that myelin is the primary origin of susceptibility anisotropy. Secondly, the bulk susceptibility effect was also a concern in part due to imperfect elimination of air bubbles in this type of phantoms. To control these effects, we constructed a numerical phantom that simulates the structures of packed axons in the white matter more precisely. Specifically, a cubic voxel packed with an ensemble of parallel axons was generated. The voxel had a dimension of *d* = 256 μm on all sides. The axons were aligned along the *z*-axis. The inner radius of the axon was 3.5 μm and the outer radius was 5.0 μm. The distance between two neighboring axons was 11.0 μm. The susceptibility of the axons was set to be −0.082 ppm and the susceptibility anisotropy (χ_ǁ_ − χ_⊥_) of the myelin sheath was 0.163 ppm with χ_||_ being −0.1 ppm (Li et al., 2012a). Here χ_||_ is defined as the susceptibility along the direction parallel to the axon. The susceptibility of the interstitial space was assumed to be zero as the reference. The voxel was divided into a 512 × 512 × 512 grid resulting in a grid size of 0.5 μm. A susceptibility tensor was assigned to each grid point depending on the tissue type (myelin, axon, or interstitial space). Only grid points within the myelin sheath had anisotropic tensors. The major eigenvectors of the myelin tensors were perpendicular to the long axis of the axon.

Once the susceptibility of each grid point within the voxel was assigned, the magnetic field at each grid point was computed *via* the forward Fourier relationship between susceptibility tensor and magnetic field as expressed in Equation (1). This computed magnetic field is what needed to satisfy Maxell's equations given the distribution of magnetic susceptibility and the presence of the external **B**_0_ field. The MR signal generated by the voxel was evaluated at *TE* = 20 ms and a field strength of 3T. Specifically, each grid point within the voxel (a total of 512^3^ grid points) was assumed to have the same proton density; however, each grid precessed at its own frequency corresponding to the magnetic field of its specific location. For a given external field orientation, the total signal of the voxel was computed as a complex summation of signals originating from all grid points within the voxel. A total of 10,000 field orientations were simulated. The mean frequency shift of the whole voxel was computed for each orientation by computing the phase of the summed signal. From this mean frequency shift, the AMS was computed by inverting Equation (1). Because only a single voxel was present, the second term in Equation (1), i.e., the demagnetization term was ignored when computing the AMS for each orientation.

### MRI experiments *In vivo*

A healthy adult was scanned on a 3T MRI system (GE MR750, GE Healthcare, Waukesha, Wisconsin) equipped with an 8-channel head coil and a maximal gradient strength of 50 mT/m. Diffusion weighted images were acquired using a dual-echo sequence and following parameters: FOV = 256 × 256 cm^2^, matrix size = 128 × 128 (reconstructed to 256 × 256 by zero filling), *TR* = 10.2 s, *TE* = 98.5 ms, number of slices = 73, number of diffusion directions = 30 and *b*-values of 1000 and 2500 s/mm^2^. Five images with *b* = 0 were also acquired. All images were acquired in a single run to maintain the same T2-weighting for both *b*-values.

Gradient-echo images were also acquired on the same scanner. A quadrature head coil was employed for the gradient echo imaging to allow a wider range of head orientations inside the coil. Gradient-echo images with various head orientations with respect to the main magnetic field were acquired using a standard flow-compensated 3D spoiled-gradient-recalled-echo (SPGR) sequence with the following parameters: *TE* = 40 ms, *TR* = 60 ms, flip angle = 20°, FOV = 256 × 256 × 256 mm^2^, matrix size = 128 × 128 × 128. Shimming was performed at each head orientation. A total of 16 orientations were acquired to achieve the rotation angle of −42~52° (around the anterior-posterior direction) and −43~39° (around the left-right direction). All human studies were approved by Institutional Review Board.

Image phase was unwrapped with a Laplacian-based phase unwrapping algorithm (Li et al., 2011; Wu et al., 2012) and filtered with the sphere mean value filtering, with a radius of 25 voxels with the radius decreasing toward the brain boundary (Li et al., 2011). Phase value was normalized by the TE to yield the frequency shift. AMS was first quantified at each brain orientation with respect to the main magnetic field using the algorithm for sparse linear equations and sparse least squares (LSQR) (Paige and Saunders, 1982). The multi-orientation gradient echo images were linearly registered to the non-diffusion weighted DTI images using FSL-FLIRT (Oxford Center for Functional MRI of the Brain, Oxford, UK). The resulting transformation matrix was used to rotate the phase and susceptibility maps and to determine the brain orientation with respect to the main magnetic field.

## Results

### Hot of crossing-fiber phantom

**Figure 1:**
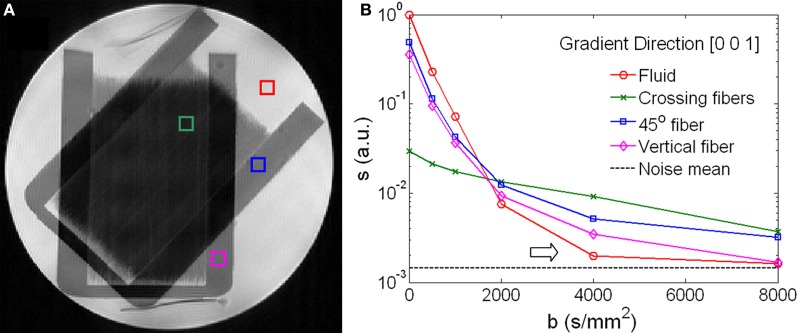
**Signal behavior of diffusion weighted images of a phantom. (A)** A T2-weighted image shows the crossing fiber structure of the phantom. Four voxels highlighted regions of fiber crossing (green), single fiber in a 45°-angle (blue), vertical fiber (magenta) and isotropic fluid (red). **(B)** Diffusion-weighted signals as a function of the *b*-values showed distinctive characteristics for the four voxels highlighted in **(A)**. In the fluid and the single fiber along the direction of [0, 0, 1], the signals approached the noise floor when *b* > 2000 s/mm^2^ (arrow).

Figure 1 shows the geometry and signal behavior in the diffusion phantom. The phantom consisted of two sets of capillaries intersecting at 45° as illustrated by the T2-weighted image (Figure 1A). Four voxels were selected within the phantom. One voxel situated in the intersecting area represented by a green box; a second voxel situated in an area with a single fiber orientation (45°) represented by a blue box; a third voxel situated in a area with vertically oriented fibers represented by a magenta box; a fourth voxel situated in the surrounding liquid represented by a red box (Figure 1A). The SNR at *b* = 0 was 2536, 1134, and 75 respectively for liquid, single fibers, and crossing fibers. The diffusion signal in these four voxels was plotted as a function of the *b*-value along the diffusion encoding direction of [0, 0, 1] (Figure 1B). The signal curves were displayed in logarithmic scale and coded with the color as the voxels were labeled in Figure 1A. In the fluid, the signal decayed exponentially up to *b* = 2000 s/mm^2^. At *b* = 4000 s/mm^2^, the signal was approaching the noise level and the decay curve bent upward (arrow in Figure 1B), giving a false impression of non-Gaussian diffusion. Similar behavior was observed in the single fiber voxel. In the voxel of crossing fibers, the signal decay curve bent upward rather early at *b* = 1000 s/mm^2^ while the signal strength was still more than 10 times higher than the noise level.

**Figure 2:**
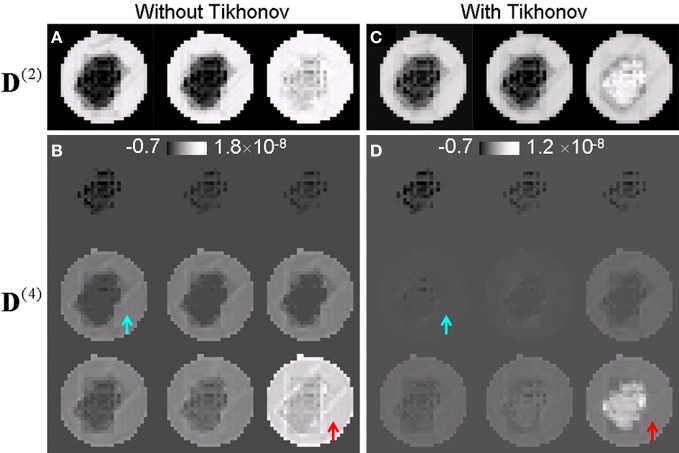
**Higher-order tensors estimated with and without Tikhonov regularization. (A,B)** Eigenvalues of the second and the fourth order tensor estimated without regularization. The fourth order tensor was overestimated for the fluid (arrows). **(C,D)** Corresponding eigenvalues of the second and the fourth order tensor estimated with regularization. The contrast between regions of fiber crossing and surrounding areas was significantly improved.

Figure 2 compares the higher order tensors estimated with and without Tikhonov regularization. Figures 2A,C compares the three eigenvalues of the second order tensor while Figures 2B,D compares the nine eigenvalues of the fourth order tensor. Without Tikhonov regularization, the fourth order tensor elements were severely overestimated in the liquid region. Without regularization, the eigenvalues of the fourth order tensor in the liquid was similar or slightly larger than those of the crossing fibers (Figure 2B) which is not physical as the diffusion in the liquid is expected to be largely Gaussian. The application of regularization suppressed the fourth order tensor in the liquid (arrows in Figure 2D). Note that the eigenvalues of the fourth order tensor can be negative as the tensor is not restricted to be positive definite in general. Similar results were observed *in vivo*. ^R3^

### Diffusion PDF and AMS orientation distribution in crossing fibers

**Figure 3:**
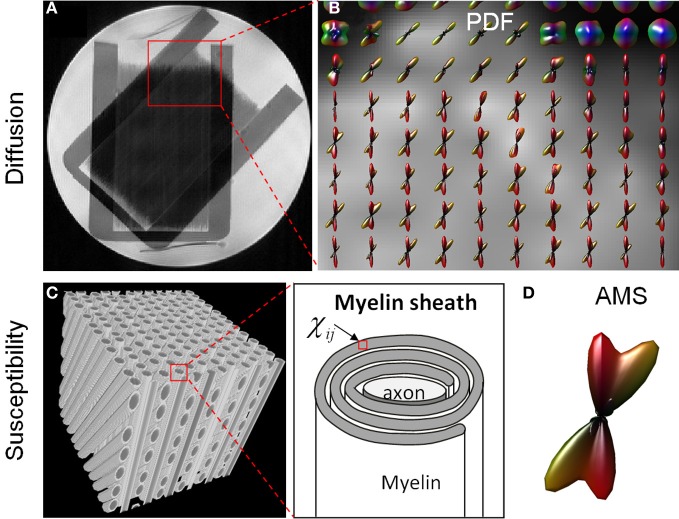
**Comparison of HOT (A,B) and AMS (C,D) glyphs in fiber crossing. (A)** A T2-weighted image illustrated a region of complex fiber structures with both single fibers and crossing fibers (red box). **(B)** The PDF glyphs over this ROI reconstructed by HOT agreed with the known phantom structure. **(C)** An illustration of myelinated axons crossing at 45°. Each voxel in the myelin sheath contained an anisotropic susceptibility tensor. **(D)** The distribution of AMS depicted a 45° crossing.

Figure 3 compares the fiber crossing reconstructed by higher-order diffusion tensors and that by the apparent magnetic susceptibility. From the PDF glyphs, regions of fiber crossing and regions of single fibers were readily identifiable (Figure 3B). These regions matched exactly as shown in the anatomical T2-weighted image (Figure 3A). The crossing angle measured based on the PDF glyphs also agreed with the true crossing angle of 45°. AMS profile was evaluated on a simulated fiber crossing in a cubic voxel. Figure 3C illustrates the arrangement of axons within the voxel. Each fiber consists of an axon and insulating myelin sheath which has an anisotropic susceptibility tensor (Figure 3B). The orientation distribution of the calculated AMS demonstrated two components intersecting at 45° consistent with the underlying geometry.

### Human brain *in vivo*

**Figure 4:**
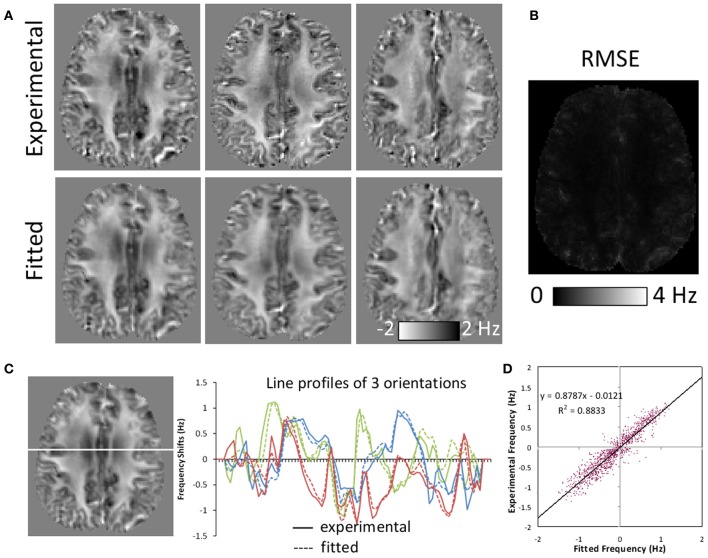
**Comparison of frequency maps measured experimentally and those fitted with susceptibility tensors. (A)** Comparison of representative experimental and fitted frequency maps along three orientations. Fitted maps were slightly smoothed but showing similar contrast. **(B)** A representative root-mean-squared-error (RMSE) map between the experimental and fitted frequency along one orientation. Significant errors were only observed around tissue boundaries. **(C)** Line profiles of experimental and fitted frequency maps for the same three orientations as in **(A)**. **(D)** Experimental and fitted frequency values along the line shown in **(C)** demonstrating high correlation.

The SNR of the diffusion-weighted brain images at *b* = 0 was 60 for the parenchyma on average. The SNR of the gradient-echo images was 34.8 for the parenchyma. Figure 4 illustrates the quality of fitting based on the susceptibility tensor model. The expected frequency maps based on the fitted susceptibility tensors are slightly smoothed but demonstrate similar contrast as the corresponding experimental frequency maps (Figure 4A). The root-mean-squared-error (RMSE) map for a representative orientation shows elevated error around tissue boundaries, but demonstrating small errors in most areas of the brain (Figure 4B). This consistency is further illustrated by three line profile plots (Figure 4C) and a correlation analysis (Figure 4D). Small deviations between experimental and fitted values were observed in the line profiles. Deviations within the white matter regions indicate potential inadequacy of the tensor model that may be induced by more complicated fiber structures. Overall, the correlation coefficient was high with *R*^2^ = 0.8833.

**Figure 5:**
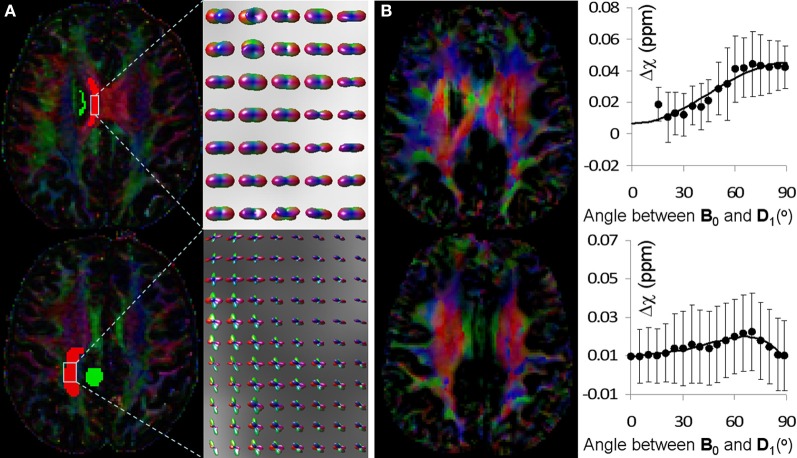
**Comparison of fiber orientations reconstructed by HOT and the orientation dependence of AMS. (A)** Color coded FA map reconstructed by DTI (left) and fiber orientations of selected ROI reconstructed by HOT. The color scheme was: red representing left-right, green representing anterior-posterior and blue representing superior-inferior. In the top row, fiber orientations were primarily unidirectional in the left-right direction; in the bottom row, fiber crossings were extensive and clearly visible in the PDF glyphs. **(B)** STI eigenvector orientation map (left) and corresponding AMS plot as a function of fiber angle over the same ROI in **(A)**. AMS was calculated using the adjacent green ROI as a reference to reduce global heterogeneity of magnetic susceptibility. Fiber angles were computed as the angles between DTI fiber orientations and the B_0_ field (superior-inferior).

Figure 5 shows the fiber orientations reconstructed by HOT and the corresponding behavior of AMS as a function of fiber angle determined by DTI. In the top row of Figure 5A, the PDF glyphs showed parallel fibers in a selected region of interest (ROI); in the bottom row of Figure 5A, the PDF glyphs of the selected ROI showed extensive fiber crossings. In the ROI with parallel fibers, AMS followed a sine-squared relationship with the fiber angle (top row of Figure 5B). The fiber angle was computed as the angle between the major eigenvector of the diffusion tensor and the B_0_ field. The principal orientations provided by STI showed resemblance to those by DTI in this region; however, significant differences also existed, consistent with previous reports (Li et al., 2012a,b). These discrepancies were thought to be caused by residual field gradients. ^*R*3^ On the other hand, in the ROI with fiber crossings, AMS no longer followed the sine-squared relationship (bottom row of Figure 5B).

## Discussion

The discovery of anisotropic diffusion has led to an explosive development of diffusion based MRI techniques for probing the microstructure of brain white matter and brain connectivity. Until very recently, diffusion MRI, in particular diffusion tensor MRI, had been only way capable of imaging white matter fiber orientations *in vivo* and non-invasively. The findings of anisotropic magnetic susceptibility in the white matter may eventually provide an alternative method. In this study, we explored for the first time the behavior of AMS in the case of fiber crossing. The behavior was investigated both in phantoms and *in vivo*. The relationship between complex AMS profile and non-Gaussian diffusion in these situations were evaluated based on the high-order tensor model of diffusion.

Previous studies have shown that in the case of parallel fibers, the orientation dependence of magnetic susceptibility can be described by a rank-2 tensor (Liu, 2010). The orientation of the major eigenvector of the susceptibility tensor was found to be aligned with the underlying fibers (Liu, 2010; Li et al., 2012a,b). Initial fiber tracking based on STI demonstrated similar tracts with DTI tractography in major white matter tracks of mouse brains *ex vivo* (Liu et al., 2012). However, it is also known that the eigenvector orientations of susceptibility tensors may not completely agree with those of diffusion tensors *in vivo*. Part of the mismatch has been attributed to residual field gradients and imperfect image registration (Li et al., 2012a,b). Another important source of the mismatch could be the complex fiber architecture of white matter fibers that deviate from the simple model geometry of parallel axons. The current study extended the analysis to cases of crossing fibers. In the phantom of 45° crossing, the angular distribution of AMS did not show an ellipsoidal shape. Rather, extremal AMS was found in directions along the fibers. In brain white matter of mainly parallel fibers such as the corpus callosum, AMS follow the typical sine-squared relationship as a function of fiber angles with respect to the main field. In regions of fiber crossing, AMS no longer followed this simple relationship. Although we were not able to illustrate the orientation distribution of AMS in 3D due to the limited number of orientations available, the observed deviation from the sine-squared relationship clearly indicated the existence of microstructures that differed from parallel fibers.

These behaviors of AMS are reminiscent of that of ADC in the situation of non-Gaussian diffusion. To explore whether the observed behavior of AMS indeed corresponded to the characteristics of crossing fibers, we reconstructed the underlying fibers with the higher order tensor model of diffusion. Diffusion tensors up to the fourth order were computed for both the phantom and the human brain. To control the bias caused by the non-zero noise floor in Rician distributed diffusion-weighted signals, we applied a Tikhonov regularization based on the mean diffusion coefficient. If the mean diffusion coefficient is larger than 1.5 × 10^−3^ mm^2^/s, then the corresponding higher order tensor elements are penalized. This regularization effectively suppressed the otherwise artificially high higher order tensor elements in the liquid of the phantom and the CSF of the brain. In addition, the tensors of fiber crossings were more accurately estimated as evidenced by the enhanced contrast of areas of fiber crossings. Alternatively, the issue of Rician distributed signal may also be resolved with a maximum likelihood estimator once the statistics of the Rician signal is estimated (Sijbers and den Dekker, 2004; Basu et al., 2006). However, this alternative strategy is generally computationally expensive over a 3D volume of images. The Tikhonov approach was efficient without any significant increase of computational burden. The corresponding PDF reconstructed based on these tensors accurately matched the fiber crossing features in the phantom. In the human brain, the PDF showed single fiber orientations in the corpus callosum which explained the sine-squared behavior of AMS. Away from these simple structures, the corresponding PDF clearly illustrated the underlying fiber crossings which explained the deviation of AMS from the sine-squared behavior.

Our preliminary investigation of magnetic susceptibility behavior in crossing fibers indicated that magnetic susceptibility may provide sufficient information for resolving fiber crossings in certain cases. To fully capture the structural characteristics, it appeared that more orientational sampling would be necessary. This was demonstrated on a simulated voxel of crossing fibers. The complex signal of the voxel was evaluated at multiple field orientations. It was found that the orientation distribution of AMS was indicative of the fiber structure. The associated challenge obviously is the increased scan time and necessity to change the position of the brain with respect to the main magnetic field. For example, while we showed that AMS no longer follows a sine-squared relationship *in vivo* in the case of fiber crossing (Figure 5), the limited number of orientations did not allow us to plot the 3D distribution of AMS. With the available number of orientations, we were able to detect fiber crossing but not able to determine the multiple fiber orientations. ^R3^ Currently, this challenge is substantial *in vivo* but the technique can be readily applied *ex vivo* or in some cases of small animal imaging. When a sufficient number of orientations are provided, the structural information may be decoded by analyzing the orientation distribution of the AMS. A simple graphic display of AMS in 3D may be sufficient to identify the underlying fiber orientations. More sophisticated or quantitative method may also be developed such as with the use of spherical harmonic functions, higher order tensors or the recently proposed multipole tensors (Liu and Li, 2013).

Further studies are needed to determine whether combining susceptibility and diffusion information may provide a more complete characterization of tissue microstructure. It has been shown that susceptibility anisotropy originates from two properties of the white matter. One is the diamagnetic property of the lipid molecules of the myelin bilayer; the other is the ordered arrangement of the lipid molecules around the axons (Lee et al., 2010; Liu, 2010; Li et al., 2012a). On the other hand, diffusion anisotropy appeared to originate primarily from the axon itself. When the myelin is removed from the white matter, for example, in the shiverer mouse, diffusion anisotropy is reduced by about 10–15% while susceptibility contrast and susceptibility anisotropy of the white matter largely disappeared (Liu et al., 2011). The information provided by susceptibility and that by diffusion are thus complementary. Probing the microstructure of white matter simultaneously with susceptibility and diffusion imaging may be beneficial for differentiating abnormalities that are due to axon degeneration and demyelination. Susceptibility imaging may be advantageous in situations when high spatial resolution is required as the resolution of diffusion-weighted images has largely been limited to be around 2 × 2 × 2 mm^3^. Susceptibility images, on the other hand, can be readily acquired at much higher spatial resolution and they are inherently three dimensional. Susceptibility contrast is also inherently advantageous at ultra-high field strength (≥7T) due to increased phase contrast (Duyn et al., 2007), low specific absorption rate and minimal sensitivity to B1 field inhomogeneity. The application of diffusion weighted images at 7T has been significantly hampered by increased RF heating and B1 inhomogeneity. Integrating susceptibility and diffusion contrast at ultra-high field strength may therefore become especially beneficial.

In conclusion, we showed that magnetic susceptibility may be used to probe complex white matter structures in the presence of fiber crossings. When the tissue contains only parallel fibers, the corresponding susceptibility can be characterized by a rank-2 tensor; when the tissue contains multiple fiber orientations, the behavior of magnetic susceptibility no longer follows the sine-squared relationship and cannot be characterized by a single rank-2 tensor. The orientation information provided by the magnetic susceptibility was consistent with that by HOT diffusion model. Susceptibility and diffusion together may provide a more complete characterization of white matter microstructure.

### Conflict of interest statement

The authors declare that the research was conducted in the absence of any commercial or financial relationships that could be construed as a potential conflict of interest.

## References

[B1] Alexander D. C. Barker G. J. Arridge S. R. (2002). Detection and modeling of non-Gaussian apparent diffusion coefficient profiles in human brain data. Magn. Reson. Med. 48, 331–340 10.1002/mrm.10209 12210942

[B2] Basser P. J. Pajevic S. (2007). Spectral decomposition of a 4th-order covariance tensor: applications to diffusion tensor MRI. Sig. Process. 87, 220–236

[B3] Basser P. J. Mattiello J. LeBihan D. (1994). MR diffusion tensor spectroscopy and imaging. Biophys. J. 66, 259–267 10.1016/S0006-3495(94)80775-1 8130344 PMC1275686

[B4] Basser P. J. Pajevic S. Pierpaoli C. Duda J. Aldroubi A. (2000). *In vivo* fiber tractography using DT-MRI data. Magn. Reson. Med. 44, 625–632 10.1002/1522-2594(200010)44:4<625::AID-MRM17>3.0.CO;2-O 11025519

[B5] Basu S. Fletcher T. Whitaker R. (2006). Rician noise removal in diffusion tensor MRI. Med. Image Comput. Comput. Assist. Interv. 9(Pt 1), 117–125 17354881 10.1007/11866565_15

[B6] Beaulieu C. (2002). The basis of anisotropic water diffusion in the nervous system – a technical review. NMR Biomed. 15, 435–455 10.1002/nbm.782 12489094

[B7] Browaeys J. T. Chevrot S. (2004). Decomposition of the elastic tensor and geophysical applications. Geophys. J. Int. 159, 667–678

[B8] Conturo T. E. Lori N. F. Cull T. S. Akbudak E. Snyder A. Z. Shimony J. S. (1999). Tracking neuronal fiber pathways in the living human brain. Proc. Natl. Acad. Sci. U.S.A. 96, 10422–10427 10.1073/pnas.96.18.10422 10468624 PMC17904

[B9] Duyn J. H. van Gelderen P. Li T. Q. de Zwart J. A. Koretsky A. P. Fukunaga M. (2007). High-field MRI of brain cortical substructure based on signal phase. Proc. Natl. Acad. Sci. U.S.A. 104, 11796–11801 10.1073/pnas.0610821104 17586684 PMC1913877

[B10] Frank L. R. (2001). Anisotropy in high angular resolution diffusion-weighted MRI. Magn. Reson. Med. 45, 935–939 10.1002/mrm.1125 11378869

[B11] Frank L. R. (2002). Characterization of anisotropy in high angular resolution diffusion-weighted MRI. Magn. Reson. Med. 47, 1083–1099 10.1002/mrm.10156 12111955

[B12] Jensen J. H. Helpern J. A. Ramani A. Lu H. Kaczynski K. (2005). Diffusional kurtosis imaging: the quantification of non-gaussian water diffusion by means of magnetic resonance imaging. Magn. Reson. Med. 53, 1432–1440 10.1002/mrm.20508 15906300

[B13] King M. D. Houseman J. Roussel S. A. van Bruggen N. Williams S. R. Gadian D. G. (1994). q-space imaging of the brain. Magn. Reson. Med. 32, 707–713 7869892 10.1002/mrm.1910320605

[B14] Lee J. Shmueli K. Fukunaga M. van Gelderen P. Merkle H. Silva A. C. (2010). Sensitivity of MRI resonance frequency to the orientation of brain tissue microstructure. Proc. Natl. Acad. Sci. U.S.A. 107, 5130–5135 10.1073/pnas.0910222107 20202922 PMC2841900

[B15] Li W. Wu B. Liu C. (2011). Quantitative susceptibility mapping of human brain reflects spatial variation in tissue composition. Neuroimage 55, 1645–1656 10.1016/j.neuroimage.2010.11.088 21224002 PMC3062654

[B16] Li W. Wu B. Avram A. V. Liu C. (2012a). Magnetic susceptibility anisotropy of human brain *in vivo* and its molecular underpinnings. Neuroimage 59, 2088–2097 10.1016/j.neuroimage.2011.10.038 22036681 PMC3254777

[B17] Li X. Vikram D. S. Lim I. A. Jones C. K. Farrell J. A. van Zijl P. C. (2012b). Mapping magnetic susceptibility anisotropies of white matter *in vivo* in the human brain at 7T. Neuroimage 62, 314–330 10.1016/j.neuroimage.2012.04.042 22561358 PMC3392309

[B18] Lin C. P. Wedeen V. J. Chen J. H. Yao C. Tseng W. Y. (2003). Validation of diffusion spectrum magnetic resonance imaging with manganese-enhanced rat optic tracts and *ex vivo* phantoms. Neuroimage 19, 482–495 10.1016/S1053-8119(03)00154-X 12880782

[B19] Liu C. (2010). Susceptibility tensor imaging. Magn. Reson. Med. 63, 1471–1477 10.1002/mrm.22482 20512849 PMC2990786

[B20] Liu C. Li W. (2013). Imaging neural architecture of the brain based on its multipole magnetic response. Neuroimage 67, 193–202 10.1016/j.neuroimage.2012.10.050 23116817 PMC3640835

[B21] Liu C. Bammer R. Acar B. Moseley M. E. (2004). Characterizing non-Gaussian diffusion by using generalized diffusion tensors. Magn. Reson. Med. 51, 924–937 10.1002/mrm.20071 15122674

[B22] Liu C. Bammer R. Acar B. Moseley M. E. (2003a). Generalized diffusion tensor imaging (GDTI) using higher order tensor (HOT) statistics, in Proceedings of the 11th Annual Meeting of ISMRM (Toronto, ON), 242. 10.1002/mrm.22192

[B23] Liu C. Bammer R. Moseley M. E. (2003b). Generalized diffusion tensor imaging (GDTI): a method for characterizing and imaging diffusion anisotropy caused by non-gaussian diffusion. Isr. J. Chem. 145–154

[B24] Liu C. Li W. Johnson G. A. Wu B. (2011). High-field (9.4 T) MRI of brain dysmyelination by quantitative mapping of magnetic susceptibility. Neuroimage 56, 930–938 10.1016/j.neuroimage.2011.02.024 21320606 PMC3085608

[B25] Liu C. Li W. Wu B. Jiang Y. Johnson G. A. (2012). 3D fiber tractography with susceptibility tensor imaging. Neuroimage 59, 1290–1298 10.1016/j.neuroimage.2011.07.096 21867759 PMC3235503

[B26] Miehe C. (1993). Computation of isotropic tensor functions. Commun. Numer. Methods Eng. 9, 889–896 10.1063/1.2812280 18205453

[B27] Mori S. Crain B. J. Chacko V. P. van Zijl P. C. (1999). Three-dimensional tracking of axonal projections in the brain by magnetic resonance imaging. Ann. Neurol. 45, 265–269 9989633 10.1002/1531-8249(199902)45:2<265::aid-ana21>3.0.co;2-3

[B28] Moseley M. E. Cohen Y. Kucharczyk J. Mintorovitch J. Asgari H. S. Wendland M. F. (1990). Diffusion-weighted MR imaging of anisotropic water diffusion in cat central nervous system. Radiology 176, 439–445 2367658 10.1148/radiology.176.2.2367658

[B29] Muti D. Bourennane S. (2005). Multiway filtering based on fourth-order cumulants. EURASIP J. Appl. Sig. Process. 2005, 1147–1158

[B30] Nemat-Nasser S. Hori M. (1999). Micromechanics: Overall Properties of Heterogeneous Materials. Amsterdam; New York: Elsevier

[B31] Ozarslan E. Mareci T. H. (2003). Generalized diffusion tensor imaging and analytical relationships between diffusion tensor imaging and high angular resolution diffusion imaging. Magn. Reson. Med. 50, 955–965 10.1002/mrm.10596 14587006

[B32] Paige C. C. Saunders M. A. (1982). Lsqr – an algorithm for sparse linear-equations and sparse least-squares. ACM Trans. Math. Softw. 8, 43–71 10.1016/j.cmpb.2011.12.002 22325240

[B33] Schweser F. Sommer K. Deistung A. Reichenbach J. R. (2012). Quantitative susceptibility mapping for investigating subtle susceptibility variations in the human brain. Neuroimage 62, 2083–2100 10.1016/j.neuroimage.2012.05.067 22659482

[B34] Sijbers J. den Dekker A. J. (2004). Maximum likelihood estimation of signal amplitude and noise variance from MR data. Magn. Reson. Med. 51, 586–594 10.1002/mrm.10728 15004801

[B35] Song S. K. Sun S. W. Ramsbottom M. J. Chang C. Russell J. Cross A. H. (2002). Dysmyelination revealed through MRI as increased radial (but unchanged axial) diffusion of water. Neuroimage 17, 1429–1436 10.1006/nimg.2002.1267 12414282

[B36] Tuch D. S. (2004). Q-ball imaging. Magn. Reson. Med. 52, 1358–1372 10.1002/mrm.20279 15562495

[B37] Tuch D. S. Reese T. G. Wiegell M. R. Makris N. Belliveau J. W. Wedeen V. J. (2002). High angular resolution diffusion imaging reveals intravoxel white matter fiber heterogeneity. Magn. Reson. Med. 48, 577–582 10.1002/mrm.10268 12353272

[B38] Wedeen V. J. Wang R. P. Schmahmann J. D. Benner T. Tseng W. Y. Dai G. (2008). Diffusion spectrum magnetic resonance imaging (DSI) tractography of crossing fibers. Neuroimage 41, 1267–1277 10.1016/j.neuroimage.2008.03.036 18495497

[B39] Wu B. Li W. Guidon A. Liu C. (2012). Whole brain susceptibility mapping using compressed sensing. Magn. Reson. Med. 67, 137–147 10.1002/mrm.23000 21671269 PMC3249423

[B40] Zucker U. Schulz H. (1982). Statistical approaches for the treatment of anharmonic motion in crystals. I. A comparison of the most frequently used formalisms of anharmonic thermal vibrations. Acta Crystallogr. Sect. A 38, 563–568

